# Machine learning for neuroimaging with scikit-learn

**DOI:** 10.3389/fninf.2014.00014

**Published:** 2014-02-21

**Authors:** Alexandre Abraham, Fabian Pedregosa, Michael Eickenberg, Philippe Gervais, Andreas Mueller, Jean Kossaifi, Alexandre Gramfort, Bertrand Thirion, Gaël Varoquaux

**Affiliations:** ^1^Parietal Team, INRIA Saclay-Île-de-FranceSaclay, France; ^2^Neurospin, I^2^ BM, DSV, CEAGif-Sur-Yvette, France; ^3^Institute of Computer Science VI, University of BonnBonn, Germany; ^4^Department of Computing, Imperial College LondonLondon, UK; ^5^Institut Mines-Telecom, Telecom ParisTech, CNRS LTCIParis, France

**Keywords:** machine learning, statistical learning, neuroimaging, scikit-learn, Python

## Abstract

Statistical machine learning methods are increasingly used for neuroimaging data analysis. Their main virtue is their ability to model high-dimensional datasets, e.g., multivariate analysis of activation images or resting-state time series. Supervised learning is typically used in *decoding* or *encoding* settings to relate brain images to behavioral or clinical observations, while unsupervised learning can uncover hidden structures in sets of images (e.g., resting state functional MRI) or find sub-populations in large cohorts. By considering different functional neuroimaging applications, we illustrate how scikit-learn, a Python machine learning library, can be used to perform some key analysis steps. Scikit-learn contains a very large set of statistical learning algorithms, both supervised and unsupervised, and its application to neuroimaging data provides a versatile tool to study the brain.

## 1. Introduction

Interest in applying statistical machine learning to neuroimaging data analysis is growing. Neuroscientists use it as a powerful, albeit complex, tool for statistical inference. The tools are developed by computer scientists who may lack a deep understanding of the neuroscience questions. This paper aims to fill the gap between machine learning and neuroimaging by demonstrating how a general-purpose machine-learning toolbox, scikit-learn, can provide state-of-the-art methods for neuroimaging analysis while keeping the code simple and understandable by both worlds. Here, we focus on software; for a more conceptual introduction to machine learning methods in fMRI analysis, see Pereira et al. (2009) or Mur et al. (2009), while Hastie et al. (2001) provides a good reference on machine learning. We discuss the use of the scikit-learn toolkit as it is a reference machine learning tool and has and a variety of algorithms that is matched by few packages, but also because it is implemented in Python, and thus dovetails nicely in the rich neuroimaging Python ecosystem.

This paper explores a few applications of statistical learning to resolve common neuroimaging needs, detailing the corresponding code, the choice of the methods, and the underlying assumptions. We discuss not only prediction scores, but also the interpretability of the results, which leads us to explore the internal model of various methods. Importantly, the GitHub repository of the paper^1^ provides complete scripts to generate figures. The scope of this paper is not to present a neuroimaging-specific library, but rather code patterns related to scikit-learn. However, the nilearn library—http://nilearn.github.io—is a software package under development that seeks to simplify the use of scikit-learn for neuroimaging. Rather than relying on an immature and black-box library, we prefer here to unravel simple and didactic examples of code that enable readers to build their own analysis strategies.

The paper is organized as follows. After introducing the *scikit-learn* toolbox, we show how to prepare the data to apply *scikit-learn* routines. Then we describe the application of *supervised learning* techniques to learn the links between brain images and stimuli. Finally we demonstrate how *unsupervised learning* techniques can extract useful structure from the images.

## 2. Our tools: scikit-learn and the python ecosystem

### 2.1. Basic scientific python tools for the neuroimager

With its mature scientific stack, Python is a growing contender in the landscape of neuroimaging data analysis with tools such as Nipy (Millman and Brett, 2007) or Nipype (Gorgolewski et al., 2011). The scientific Python libraries used in this paper are:

- **NumPy**: Provides the ndarray data type to python, an efficient *n*-dimensional data representation for array-based numerical computation, similar to that used in Matlab (Van Der Walt et al., 2011). It handles efficient array persistence (input and output) and provides basic operations such as dot product. Most scientific Python libraries, including scikit-learn, use NumPy arrays as input and output data type.
- **SciPy**: Higher level mathematical functions that operate on ndarrays for a variety of domains including linear algebra, optimization and signal processing. SciPy is linked to compiled libraries to ensure high performances (BLAS, Arpack, and MKL for linear algebra and mathematical operations). Together, NumPy and SciPy provide a robust scientific environment for numerical computing and they are the elementary bricks that we use in all our algorithms.
- **Matplotlib**: A plotting library tightly integrated into the scientific Python stack (Hunter, 2007). It offers publication-quality figures in different formats and is used to generate the figures in this paper.
- **Nibabel**: To access data in neuroimaging file formats. We use it at the beginning of all our scripts.

### 2.2. Scikit-learn and the machine learning ecosystem

Scikit-learn (Pedregosa et al., 2011) is a general purpose machine learning library written in Python. It provides efficient implementations of state-of-the-art algorithms, accessible to non-machine learning experts, and reusable across scientific disciplines and application fields. It also takes advantage of Python interactivity and modularity to supply fast and easy prototyping. There is a variety of other learning packages. For instance, in Python, PyBrain (Schaul et al., 2010) is best at neural networks and reinforcement learning approaches, but its models are fairly black box, and do not match our need to interpret the results. Beyond Python, Weka (Hall et al., 2009) is a rich machine learning framework written in Java, however, it is more oriented toward data mining.

Some higher level frameworks provides full pipeline to apply machine learning techniques to neuroimaging. PyMVPA (Hanke et al., 2009) is a Python packaging that does data preparation, loading and analysis, as well as result visualization. It performs multi-variate pattern analysis and can make use of external tools such as R, scikit-learn or Shogun (Sonnenburg et al., 2010). PRoNTo (Schrouff et al., 2013) is written in Matlab and can easily interface with SPM but does not propose many machine learning algorithms. Here, rather than full-blown neuroimaging analysis pipelines, we discuss lower-level patterns that break down how neuroimaging data is input to scikit-learn and processed with it. Indeed, the breadth of machine learning techniques in scikit-learn and the variety of possible applications are too wide to be fully exposed in a high-level interface. Note that a package like PyMVPA that can rely on scikit-learn for neuroimaging data analysis implements similar patterns behind its high-level interface.

### 2.3. Scikit-learn concepts

In *scikit-learn*, all objects and algorithms accept input data in the form of 2-dimensional arrays of size samples × features. This convention makes it generic and domain-independent. Scikit-learn objects share a uniform set of methods that depends on their purpose: *estimators* can fit models from data, *predictors* can make predictions on new data and *transformers* convert data from one representation to another.

- **Estimator**. The *estimator* interface, the core of the library, exposes a fit method for learning model parameters from training data. All supervised and unsupervised learning algorithms (e.g., for classification, regression or clustering) are available as objects implementing this interface. Machine learning tasks such as feature selection or dimensionality reduction are also provided as estimators.
- **Predictor**. A *predictor* is an estimator with a predict method that takes an input array X_test and makes predictions for each sample in it. We denote this input parameter “X_test” in order to emphasize that predict generalizes to new data. In the case of supervised learning estimators, this method typically returns the predicted labels or values computed from the estimated model.
- **Transformer**. As it is common to modify or filter data before feeding it to a learning algorithm, some estimators, named *transformers*, implement a transform method. Preprocessing, feature selection and dimensionality reduction algorithms are all provided as transformers within the library. If the transformation can be inverted, a method called inverse_transform also exists.

When testing an estimator or setting hyperparameters, one needs a reliable metric to evaluate its performance. Using the same data for training and testing is not acceptable because it leads to overly confident model performance, a phenomenon also known as *overfitting*. Cross-validation is a technique that allows one to reliably evaluate an estimator on a given dataset. It consists in iteratively fitting the estimator on a fraction of the data, called *training set*, and testing it on the left-out unseen data, called *test set*. Several strategies exists to partition the data. For example, *k*-fold cross-validation consists in dividing (randomly or not) the samples in *k* subsets: each subset is then used once as testing set while the others *k* − 1 subsets are used to train the estimator. This is one of the simplest and most widely used cross-validation strategies. The parameter *k* is commonly set to 5 or 10. Another strategy, sometimes called Monte-Carlo cross-validation, uses many random partitions in the data.

For a given model and some fixed value of hyperparameters, the scores on the various test sets can be averaged to give a quantitative score to assess how good the model is. Maximizing this cross-validation score offers a principled way to set hyperparameters and allows to choose between different models. This procedure is known as *model selection*. In *scikit-learn*, hyperparameters tuning can be conviently done with the GridSearchCV estimator. It takes as input an estimator and a set of candidate hyperparameters. Cross-validation scores are then computed for all hyperparameters combinations, possibly in parallel, in order to find the best one. In this paper, we set the regularization coefficient with grid search in section 5.

## 3. Data preparation: from MR volumes to a data matrix

Before applying statistical learning to neuroimaging data, standard preprocessing must be applied. For fMRI, this includes motion correction, slice timing correction, coregistration with an anatomical image and normalization to a common template like the MNI (Montreal Neurologic Institute) one if necessary. Reference softwares for these tasks are SPM (Friston, 2007) and FSL (Smith et al., 2004). A Python interface to these tools is available in nipype Python library (Gorgolewski et al., 2011). Below we discuss shaping preprocessed data into a format that can be fed to scikit-learn. For the machine learning settings, we need a data matrix, that we will denote *X*, and optionally a target variable to predict, *y*.

### 3.1. Spatial resampling

Neuroimaging data often come as Nifti files, 4-dimensional data (3D scans with time series at each location or voxel) along with a transformation matrix (called affine) used to compute voxel locations from array indices to world coordinates. When working with several subjects, each individual data is registered on a common template (MNI, Talairach…), hence on a common affine, during preprocessing.

Affine matrix can express data anisotropy, when the distance between two voxels is not the same depending on the direction. This information is used by algorithms relying on the spatial structure of the data, for instance the Searchlight.

SciPy routine scipy.ndimage.affine_transform can be used to perform image resampling: changing the spatial resolution of the data^2^. This is an interpolation and alters the data, that is why it should be used carefully. Downsampling is commonly used to reduce the size of data to process. Typical sizes are 2 or 3 mm resolution, but scan spatial resolution is increasing with progress in MR physics. The affine matrix can encode the scaling factors for each direction.

### 3.2. Signal cleaning

Due to its complex and indirect acquisition process, neuroimaging data often have a low signal-to-noise ratio. They contain trends and artifacts that must be removed to ensure maximum machine learning algorithms efficiency. Signal cleaning includes:

- **Detrending** removes a linear trend over the time series of each voxel. This is a useful step when studying fMRI data, as the voxel intensity itself has no meaning and we want to study its variation and correlation with other voxels. Detrending can be done thanks to SciPy (scipy.signal.detrend).
- **Normalization** consists in setting the timeseries variance to 1. This harmonization is necessary as some machine learning algorithms are sensible to different value ranges.
- **Frequency filtering** consists in removing high or low frequency signals. Low-frequency signals in fMRI data are caused by physiological mechanisms or scanner drifts. Filtering can be done thanks to a Fourier transform (scipy.fftpack.fft) or a Butterworth filter (scipy.signal.butter).

### 3.3. From 4-dimensional images to 2-dimensional array: masking

Neuroimaging data are represented in 4 dimensions: 3 spatial dimensions, and one dimension to index time or trials. Scikit-learn algorithms, on the other hand, only accept 2-dimensional samples × features matrices (see section 2.3). Depending on the setting, voxels and time series can be considered as features or samples. For example, in spatial independent component analysis (ICA), voxels are samples.

**Figure 1:**
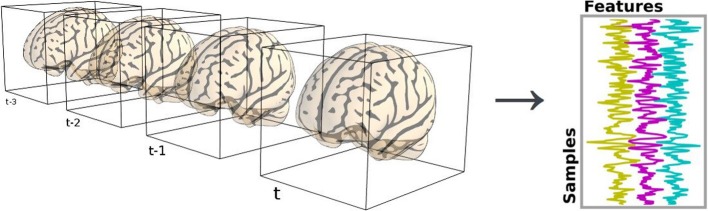
**Conversion of brain scans into 2-dimensional data**.

The reduction process from 4D-images to feature vectors comes with the loss of spatial structure (see Figure 1). It however allows to discard uninformative voxels, such as the ones outside of the brain. Such voxels that only carry noise and scanner artifacts would reduce SNR and affect the quality of the estimation. The selected voxels form a *brain mask*. Such a mask is often given along with the datasets or can be computed with software tools such as FSL or SPM.

Applying the mask is made easy by NumPy advanced indexing using boolean arrays. Two-dimensional masked data will be referred to as X to follow scikit-learn conventions:

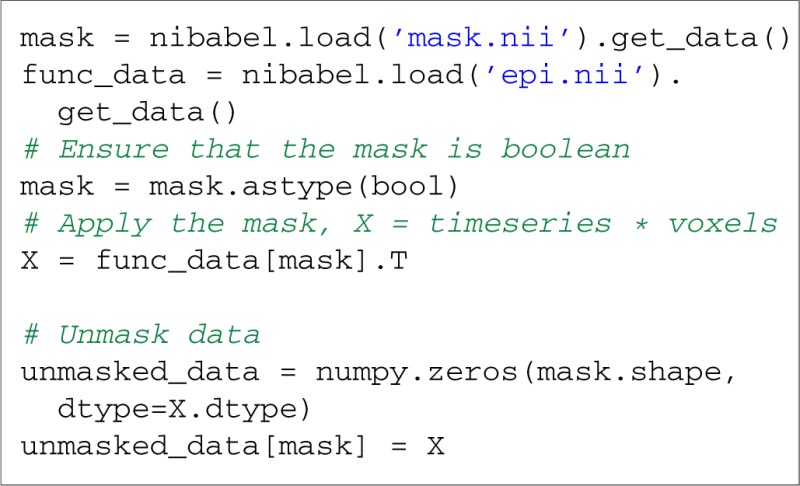



### 3.4. Data visualization

Across all our examples, voxels of interest are represented on an axial slice of the brain. Some transformations of the original matrix data are required to match matplotlib data format. The following snippet of code shows how to load and display an axial slice overlaid with an activation map. The background is an anatomical scan and its highest voxels are used as synthetic activations.

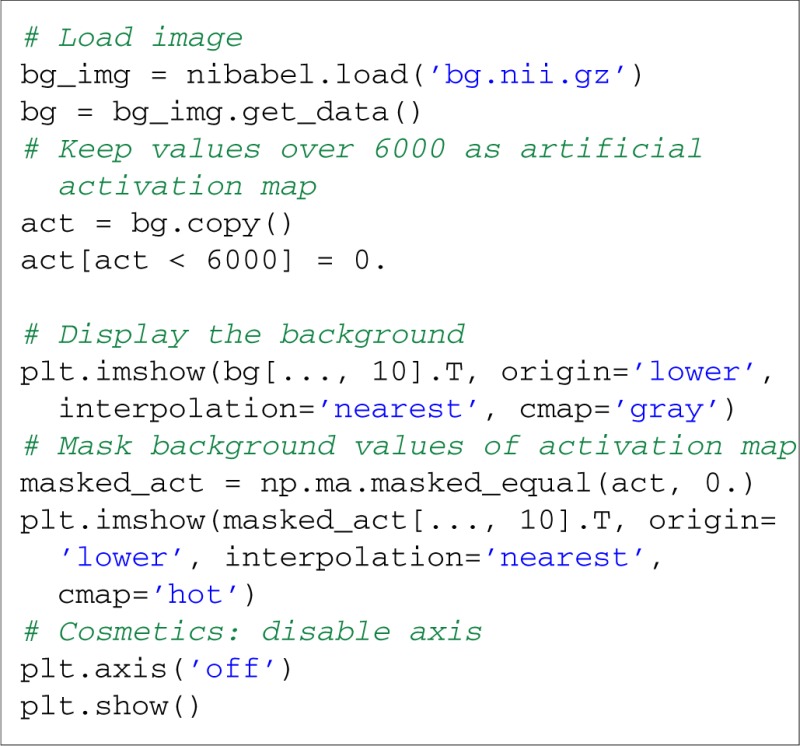



Note that a background is needed to display partial maps. Overlaying two images can be done thanks to the numpy.ma.masked_array data structure. Several options exist to enhance the overall aspect of the plot. Some of them can be found in the full scripts provided with this paper. It generally boils down to a good knowledge of Matplotlib. Note that the Nipy package provides a plot_map function that is tuned to display activation maps (a background is even provided if needed).

## 4. Decoding the mental representation of objects in the brain

In the context of neuroimaging, *decoding* refers to learning a model that predicts behavioral or phenotypic variables from brain imaging data. The alternative that consists in predicting the imaging data given external variables, such as stimuli descriptors, is called *encoding* (Naselaris et al., 2011). It is further discussed in the next section.

First, we illustrate decoding with a simplified version of the experiment presented in Haxby et al. (2001). In the original work, visual stimuli from 8 different categories are presented to 6 subjects during 12 sessions. The goal is to predict the category of the stimulus presented to the subject given the recorded fMRI volumes. This example has already been widely analyzed (Hanson et al., 2004; Detre et al., 2006; O'Toole et al., 2007; Hanson and Halchenko, 2008; Hanke et al., 2009) and has become a reference example in matter of decoding. For the sake of simplicity, we restrict the example to one subject and to two categories, faces and houses.

As there is a *target* variable *y* to predict, this is a supervised learning problem. Here *y* represents the two object categories, a.k.a. *classes* in machine-learning terms. In such settings, where *y* takes discrete values the learning problem is known as *classification*, as opposed to *regression* when the variable *y* can take continuous values, such as age.

### 4.1. Classification with feature selection and linear SVM

Many classification methods are available in scikit-learn. In this example we chose to combine the use of univariate feature selection and Support Vector Machines (SVM). Such a classification strategy is simple yet efficient when used on neuroimaging data.

After applying a brain mask, the data consist of 40,000 voxels, here the features, for only 1400 volumes, here the samples. Machine learning with many more features than samples is challenging, due to the so-called *curse of dimensionality*. Several strategies exist to reduce the number of features. A first one is based on prior neuroscientific knowledge. Here one could restrict the mask to occipital areas, where the visual cortex is located. Feature selection is a second, data-driven, approach that relies on a univariate statistical test for each individual feature. Variables with high individual discriminative power are kept.

Scikit-learn offers a panel of strategies to select features. In supervised learning, the most popular feature selection method is the F-test. The null hypothesis of this test is that the feature takes the same value independently of the value of *y* to predict. In scikit-learn, sklearn.feature_selection proposes a panel of feature selection strategies. One can choose to take a percentile of the features (SelectPercentile), or a fixed number of features (SelectKBest). All these objects are implemented as transformers (see section 2.3). The code below uses the f_classif function (ANOVA F-Test) along with the selection of a fixed number of features.

**Figure 2:**
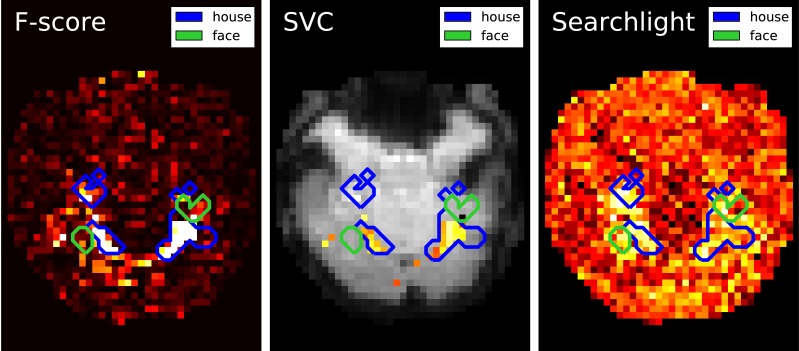
**Maps derived by different methods for face versus house recognition in the Haxby experiment—*left*: standard analysis; *center*: SVM weights after screening voxels with an ANOVA; *right*: Searchlight map**. The masks derived from standard analysis in the original paper (Haxby et al., 2001) are displayed in blue and green.

On the reduced feature set, we use a linear SVM classifier, sklearn.svm.SVC, to find the hyperplane that maximally separates the samples belonging to the different classes. Classifying a new sample boils down to determining on which side of the hyperplane it lies. With a linear kernel, the separating hyperplane is defined in the input data space and its coefficients can be related to the voxels. Such coefficients can therefore be visualized as an image (after unmasking step described in section 3.3) where voxels with high values have more influence on the prediction than the others (see Figure 2).

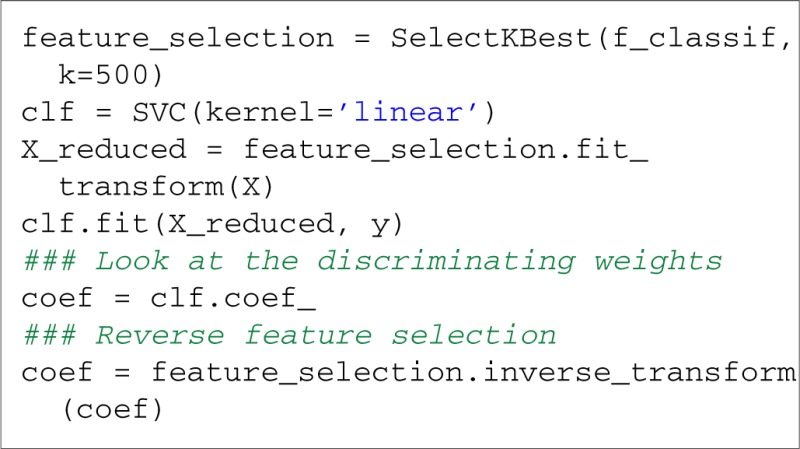



### 4.2. Searchlight

Searchlight (Kriegeskorte et al., 2006) is a popular algorithm in the neuroimaging community. It runs a predictive model on a spatial neighborhood of each voxel and tests the out-of-sample prediction performance as proxy measure of the link between the local brain activity and the target behavioral variable. In practice, it entails performing cross-validation of the model, most often an SVM, on voxels contained in balls centered on each voxel of interest. The procedure implies solving a large number of SVMs and is computationally expensive. Detailing an efficient implementation of this algorithm is beyond the scope of this paper. However, code for searchlight and to generate Figure 2 is available in the GitHub repository accompanying the paper.

### 4.3. Results

Results are shown in Figure 2: first F-score, that is standard analysis in brain mapping but also the statistic used to select features; second the SVC weights after feature selection and last the Searchlight map. Note that the voxels with larger weights roughly match for all methods and are located in the house-responsive areas as defined by the original paper. The Searchlight is more expanded and blurry than the other methods as it iterates over a ball around the voxels.

These results match neuroscientific knowledge as they highlight the high level regions of the ventral visual cortex which is known to contain category-specific visual areas. While Searchlight only gives a score to each voxel, the SVC can be used afterward to classify unseen brain scans.

Most of the final example script (haxby_decoding.py on GitHub) is for data loading and result visualization. Only five lines are needed to run a scikit-learn classifier. In addition, thanks to the scikit-learn modularity, the SVC can be easily replaced by any other classifier in this example. As all linear models share the same interface, replacing the SVC by another linear model, such as ElasticNet or LogisticRegression, requires changing only one line. Gaussian Naive Bayes is a non-linear classifier that should perform well in this case, and modifying display can be done by replacing coef_ by theta_.

## 5. Encoding brain activity and decoding images

In the previous experiment, the category of a visual stimulus was inferred from brain activity measured in the visual cortex. One can go further by inferring a direct link between the image seen by the subject and the associated fMRI data.

In the experiment of Miyawaki et al. (2008) several series of 10×10 binary images are presented to two subjects while activity on the visual cortex is recorded. In the original paper, the training set is composed of random images (where black and white pixels are balanced) while the testing set is composed of structured images containing geometric shapes (square, cross…) and letters. Here, for the sake of simplicity, we consider only the training set and use cross-validation to obtain scores on unseen data. In the following example, we study the relation between stimuli pixels and brain voxels in both directions: the reconstruction of the visual stimuli from fMRI, which is a decoding task, and the prediction of fMRI data from descriptors of the visual stimuli, which is an encoding task.

### 5.1. Decoding

In this setting, we want to infer the binary visual stimulus presented to the subject from the recorded fMRI data. As the stimuli are binary, we will treat this problem as a classification problem. This implies that the method presented here cannot be extended as-is to natural stimuli described with gray values.

In the original work, Miyawaki et al. (2008) uses a Bayesian logistic regression promoting sparsity along with a sophisticated multi-scale strategy. As one can indeed expect the number of predictive voxels to be limited, we compare the ℓ_2_ SVM used above with a logistic regression and a SVM penalized with the ℓ_1_ norm known to promote sparsity. The ℓ_1_ penalized SVM classifier compared here uses a square-hinge loss while the logistic regression uses a logit function.

Table 1 reports the performance of the different classifiers for various values of C using a fivefold cross-validation. We first observe that setting the parameter *C* is crucial as performance drops for inappropriate values of C. It is particularly true for ℓ_1_ regularized models. Both ℓ_1_ logistic regression and SVM yield similar performances, which is not surprising as they implement similar models.

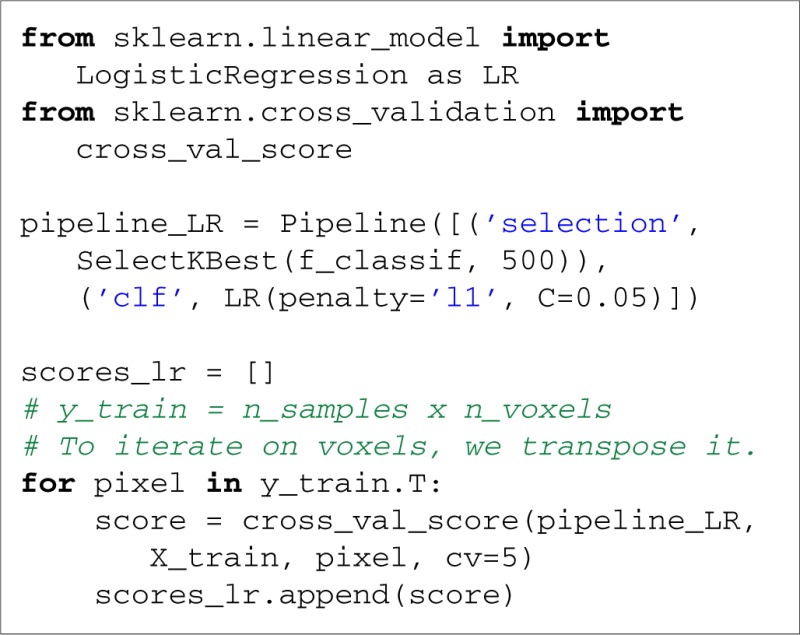



**Table 1:** **Five fold cross validation accuracy scores obtained for different values of parameter *C* (± *SD*), best scores are shown in bold**.

***C* value**	**0.0005**	**0.001**	**0.005**	**0.01**	**0.05**	**0.1**
ℓ_1_ Logistic regression	0.50 ± 0.02	0.50 ± 0.02	0.57 ± 0.13	0.63 ± 0.11	**0.70** ± 0.12	0.70 ± 0.12
ℓ_2_ Logistic regression	0.60 ± 0.11	0.61 ± 0.12	0.63 ± 0.13	0.63 ± 0.13	**0.64** ± 0.13	0.64 ± 0.13
ℓ_1_ SVM classifier (SVC)	0.50 ± 0.06	0.55 ± 0.12	0.69 ± 0.11	**0.71** ± 0.12	0.69 ± 0.12	0.68 ± 0.12
ℓ_2_ SVM classifier (SVC)	0.67 ± 0.12	**0.67** ± 0.12	0.67 ± 0.12	0.66 ± 0.12	0.65 ± 0.12	0.65 ± 0.12

### 5.2. Encoding

Given an appropriate model of the stimulus, e.g., one which can provide an approximately linear representation of BOLD activation, an encoding approach allows one to quantify for each voxel to what extent its variability is captured by the model. A popular evaluation method is the predictive (*r*^2^) score, which uses a prediction on left out data to quantify the decrease in residual norm brought about by fitting a regression function as opposed to fitting a constant. The remaining variance consists of potentially unmodeled, but reproducible signal and spurious noise.

On the Miyawaki dataset, we can observe that mere black and white pixel values can explain a large part of the BOLD variance in many visual voxels. Sticking to the notation that (*X*) represesents BOLD signal and (*y*) the stimulus, we can write an encoding model using the ridge regression estimator:

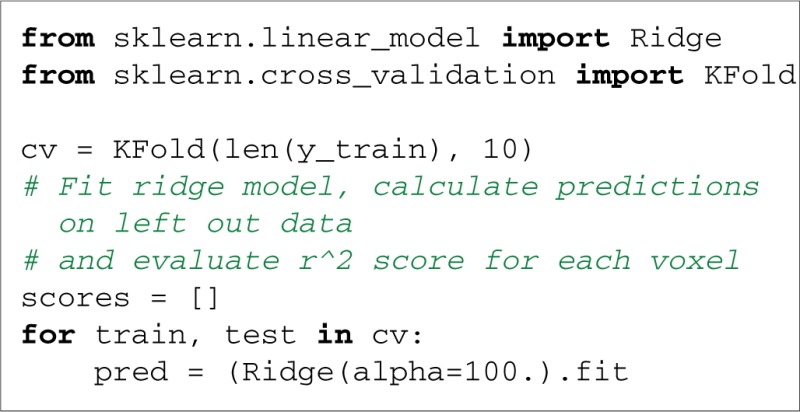

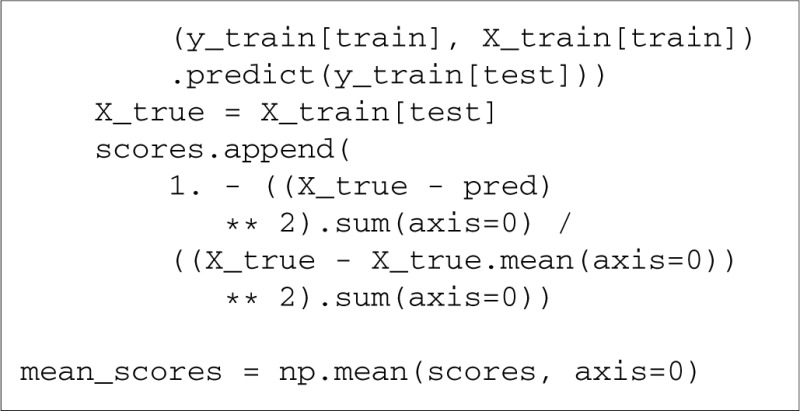





Note here that the Ridge can be replaced by a Lasso estimator, which can give better prediction performance at the cost of computation time.

#### 5.2.1. Receptive fields

Given the retinotopic structure of early visual areas, it is expected that the voxels well predicted by the presence of a black or white pixel are strongly localized in so-called population receptive fields (*prf*). This suggests that only very few stimulus pixels should suffice to explain the activity in each brain voxel of the posterior visual cortex. This information can be exploited by using a sparse linear regression—the Lasso (Tibshirani, 1996)—to find the receptive fields. Here we use the *LassoLarsCV* estimator that relies on the LARS algorithm (Efron et al., 2004) and cross-validation to set the Lasso parameter.

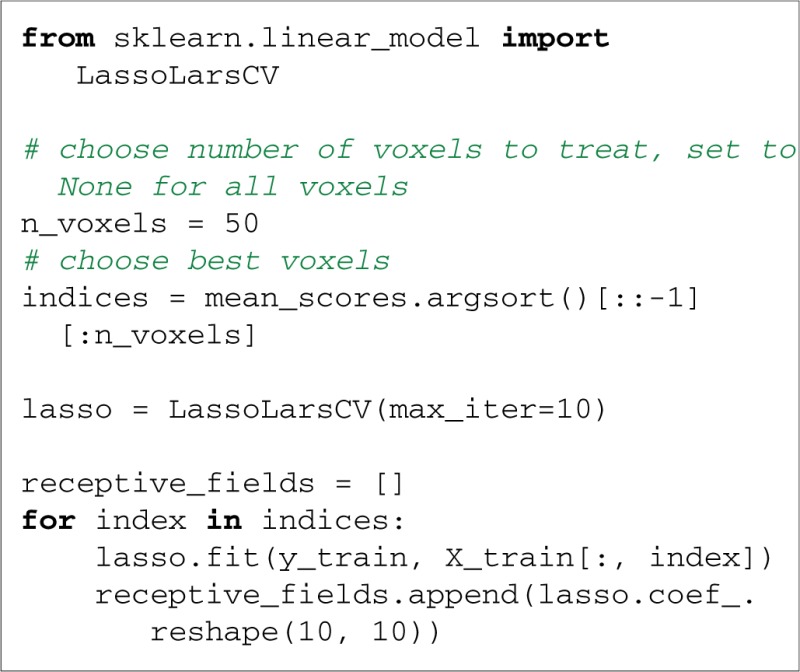



### 5.3. Results

**Figure 3:**
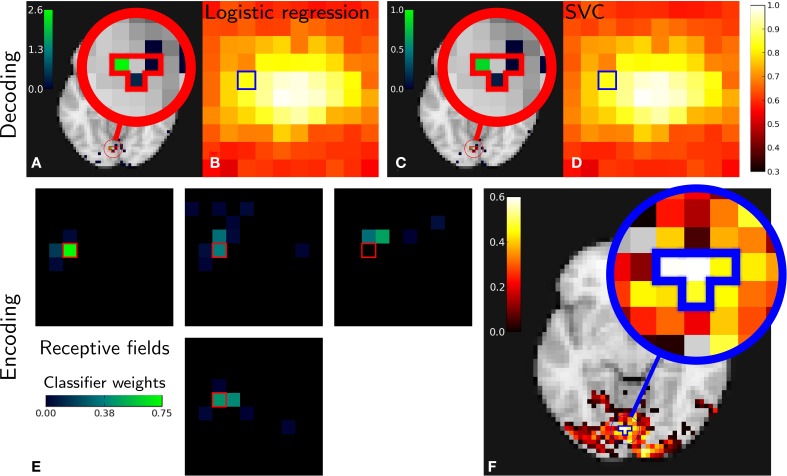
**Miyawaki results in both decoding and encoding**. Relations between one pixel and four brain voxels is highlighted for both methods. **Top: Decoding**. Classifier weights for the pixel highlighted [**(A)** Logistic regression, **(C)** SVM]. Reconstruction accuracy per pixel [**(B)** Logistic regression, **(D)** SVM]. **Bottom: Encoding**. **(E)**: receptive fields corresponding to voxels with highest scores and its neighbors. **(F)**: reconstruction accuracy depending on pixel position in the stimulus—note that the pixels and voxels highlighted are the same in both decoding and encoding figures and that encoding and decoding roughly match as both approach highlight a relationship between the same pixel and voxels.

Figure 3 gives encoding and decoding results: the relationship between a given image pixel and four voxels of interest in the brain. In decoding settings, Figures 3A,C show the classifier's weights as brain maps for both methods. They both give roughly the same results and we can see that the weights are centered in the V1 and nearby retinotopic areas. Figures 3B,D show reconstruction accuracy score using Logistic Regression (LR) and SVM (variable mean_scores in the code above). Both methods give almost identical results. As in the original work (Miyawaki et al., 2008), reconstruction is more accurate in the fovea. This is explained by the higher density of neurons dedicated to foveal representation in the primary visual area.

In encoding settings, Figure 3E shows classifiers weights for encoding, that we interpret as receptive fields. We can see that receptive fields of neighboring voxels are neighboring pixels, which is expected from retinotopy: primary visual cortex maps the visual field in a topologically organized manner.

Both encoding and decoding analysis show a link between the selected pixel and brain voxels. In the absence of ground truth, seeing that different methods come to the same conclusion comes as face validity.

## 6. Resting-state and functional connectivity analysis

Even in the absence of external behavioral or clinical variable, studying the structure of brain signals can reveal interesting information. Indeed, Biswal et al. (1995) have shown that brain activation exhibits coherent spatial patterns during rest. These correlated voxel activations form functional networks that are consistent with known task-related networks (Smith et al., 2009).

Biomarkers found via predictive modeling on resting-state fMRI would be particularly useful, as they could be applied to diminished subjects that cannot execute a specific task. Here we use a dataset containing control and ADHD (Attention Disorder Hyperactivity Disorder) patients resting state data (subjects are scanned without giving them any specific task to capture the cerebral background activity).

Resting state fMRI is unlabeled data in the sense that the brain activity at a given instant in time cannot be related to an output variable. In machine learning, this class of problems is known as unsupervised learning. To extract functional networks or regions, we use methods that group together similar voxels by comparing their time series. In neuroimaging, the most popular method is ICA that is the subject of our first example. We then show how to obtained functionally-homogeneous regions with clustering methods.

### 6.1. Independent component analysis (ICA) to extract networks

ICA is a blind source separation method. Its principle is to separate a multivariate signal into several components by maximizing their non-Gaussianity. A typical example is the *cocktail party problem* where ICA is able to separate voices from several people using signal from microphones located across the room.

#### 6.1.1. ICA in neuroimaging

ICA is the reference method to extract networks from resting state fMRI (Kiviniemi et al., 2003). Several strategies have been used to syndicate ICA results across several subjects. Calhoun et al. (2001) propose a dimension reduction (using PCA) followed by a concatenation of timeseries (used in this example). Varoquaux et al. (2010) use dimension reduction and canonical correlation analysis to aggregate subject data. Melodic (Beckmann and Smith, 2004), the ICA tool in the FSL suite, uses a concatenation approach not detailed here.

#### 6.1.2. Application

As data preparation steps, we not only center, but also detrend the time series to avoid capturing linear trends with the ICA. Applying to the resulting time series the FastICA algorithm (Hyvärinen and Oja, 2000) with scikit-learn is straightforward thanks to the transformer concept. The data matrix must be transposed, as we are using *spatial* ICA, in other words the direction considered as random is that of the voxels and not the time points. The maps obtained capture different components of the signal, including noise components as well as resting-state functional networks. To produce the figures, we extract only 10 components, as we are interested here in exploring only the main signal structures.

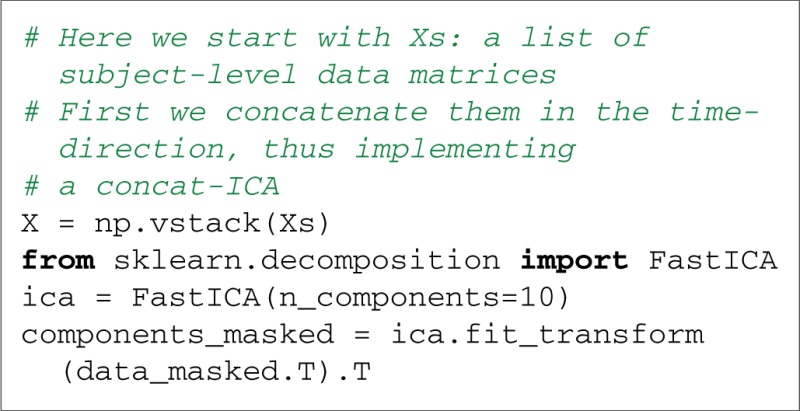



#### 6.1.3. Results

**Figure 4:**
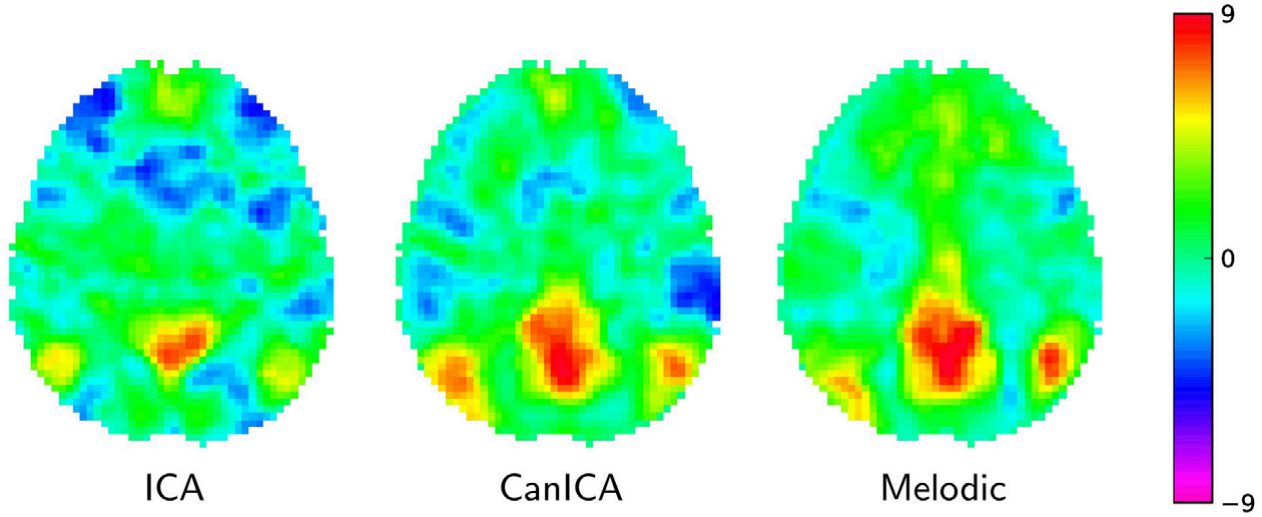
**Default mode network extracted using different approaches: *left*: the simple Concat-ICA approach detailed in this article; *middle*: CanICA, as implemented in nilearn; *right*: Melodic's concat-ICA**. Data have been normalized (set to unit variance) for display purposes.

On Figure 4 we compare a simple concat ICA as implemented by the code above to more sophisticated multi-subject methods, namely Melodic's concat ICA and CanICA—also implemented using scikit-learn although we do not discuss the code here. We display here only the default mode network as it is a well-known resting-state network. It is hard to draw conclusions from a single map but, at first sight, it seems that both CanICA and Melodic approaches are less subject to noise and give similar results.

Scikit-learn proposes several other matrix decomposition strategies listed in the module “sklearn.decomposition.” A good alternative to ICA is the dictionary learning that applies a ℓ_1_ regularization on the extracted components (Varoquaux et al., 2011). This leads to more sparse and compact components than ICA ones, which are full-brain and require thresholding.

### 6.2. Learning functionally homogeneous regions with clustering

From a machine learning perspective, a clustering method aggregates samples into groups (called clusters) maximizing a measure of similarity between samples within each cluster. If we consider voxels of a functional brain image as samples, this measure can be based on functional similarity, leading to clusters of voxels that form functionally homogeneous regions (Thirion et al., 2006).

#### 6.2.1. Approaches

Several clustering approaches exists, each one having its own pros and cons. Most require setting the number of clusters extracted. This choice depends on the application: a large number of clusters will give a more fine-grained description of the data, with a higher fidelity to the original signal, but also a higher model complexity. Some clustering approaches can make use of spatial information and yield spatially contiguous clusters, i.e., parcels. Here we will describe two clustering approaches that are simple and fast.

***6.2.1.1. Ward clustering***. uses a bottom-up hierarchical approach: voxels are progressively agglomerated together into clusters. In scikit-learn, structural information can be specified via a connectivity graph given to the Ward clustering estimator. This graph is used to allow only merges between neighboring voxels, thus readily producing contiguous parcels. We will rely on the sklearn.feature_extraction.image.grid_to_graph function to construct such a graph using the neighbor structure of an image grid, with optionally a brain mask.

***6.2.1.2. K-Means***. is a more top-down approach, seeking cluster centers to evenly explain the variance of the data. Each voxels are then assigned to the nearest center, thus forming clusters. As imposing a spatial model in K-means is not easy, it is often advisable to spatially smooth the data.

To apply the clustering algorithms, we run the common data preparation steps and produce a data matrix. As both Ward clustering and K-means rely on second-order statistics, we can speed up the algorithms by reducing the dimensionality while preserving these second-order statistics with a PCA. Note that clustering algorithms group samples and that here we want to group voxels. So if the data matrix is, as previously a (time points × voxels) matrix, we need to transpose it before running the scikit-learn clustering estimators. Scikit-learn provides a WardAgglomeration object to do this *feature agglomeration* with Ward clustering (Michel et al., 2012), but this is not the case when using K-Means.

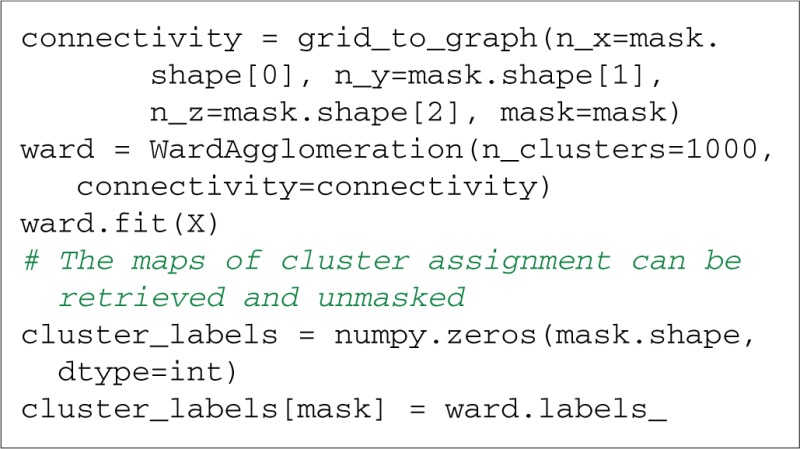



#### 6.2.2. Results

**Figure 5:**
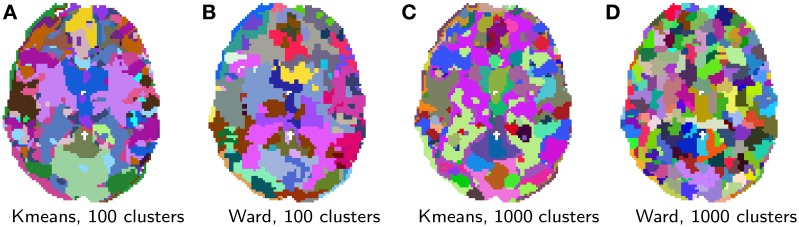
**Brain parcellations extracted by clustering**. Colors are random. **(A)** K-means, 100 clusters, **(B)** Ward, 100 clusters, **(C)** K-means, 1000 clusters, and **(D)** Ward, 1000 clusters.

Clustering results are shown in Figure 5. While clustering extracts some known large scale structure, such as the calcarine sulcus on Figure 5A, it is not guaranteed to delineate functionally specific brain regions. Rather, it can be considered as a compression, that is a useful method of summarizing information, as it groups together similar voxels. Note that, as K-means does not extract spatially-contiguous clusters, it gives a number of regions that can be much larger than the number of clusters specified, although some of these regions can be very small. On the opposite, spatially-constrained Ward directly creates regions. As it is a bottom-up process, it tends to perform best with a large number of clusters. There exist many more clustering techniques exposed in scikit-learn. Determining which is the best one to process fMRI time-series requires a more precise definition of the target application.

Ward's clustering and K-Means are among the simplest approaches proposed in the scikit-learn. Craddock et al. (2011) applied spectral clustering on neuroimaging data, a similar application is available in nilearn as an example.

## 7. Conclusion

In this paper we have illustrated with simple examples how machine learning techniques can be applied to fMRI data using the scikit-learn Python toolkit in order to tackle neuroscientific problems. Encoding and decoding can rely on supervised learning to link brain images with stimuli. Unsupervised learning can extract structure such as functional networks or brain regions from resting-state data. The accompanying Python code for the machine learning tasks is straightforward. Difficulties lie in applying proper preprocessing to the data, choosing the right model for the problem, and interpreting the results. Tackling these difficulties while providing the scientists with simple and readable code requires building a domain-specific library, dedicated to applying scikit-learn to neuroimaging data. This effort is underway in a nascent project, nilearn, that aims to facilitate the use of scikit-learn on neuroimaging data.

The examples covered in this paper only scratch the surface of applications of statistical learning to neuroimaging. The tool stack presented here shines uniquely in this regard as it opens the door to any combination of the wide range of machine learning methods present in scikit-learn with neuroimaging-related code. For instance, sparse inverse covariance can extract the functional interaction structure from fMRI time-series (Varoquaux and Craddock, 2013) using the graph-lasso estimator. Modern neuroimaging data analysis entails fitting rich models on limited data quantities. These are high-dimensional statistics problems which call for statistical-learning techniques. We hope that bridging a general-purpose machine learning tool, scikit-learn, to domain-specific data preparation code will foster new scientific advances.

### Conflict of interest statement

The authors declare that the research was conducted in the absence of any commercial or financial relationships that could be construed as a potential conflict of interest.

## Footnotes

^1^http://www.github.com/AlexandreAbraham/frontiers2013

^2^An easy-to-use implementation is proposed in nilearn.
